# Dataset of miRNA–disease relations extracted from textual data using transformer-based neural networks

**DOI:** 10.1093/database/baae066

**Published:** 2024-08-05

**Authors:** Sumit Madan, Lisa Kühnel, Holger Fröhlich, Martin Hofmann-Apitius, Juliane Fluck

**Affiliations:** Department of Bioinformatics, Fraunhofer Institute for Algorithms and Scientific Computing (SCAI), Schloss Birlinghoven, 53757 Sankt Augustin, Germany; Knowledge Management, German National Library of Medicine (ZB MED)—Information Centre for Life Sciences, Friedrich-Hirzebruch-Allee 4, Bonn 53115, Germany; Graduate School DILS, Bielefeld Institute for Bioinformatics Infrastructure (BIBI), Faculty of Technology, Bielefeld University, Postfach 10 01 31, Bielefeld, Nordrhein-Westfalen 33501, Germany; Department of Bioinformatics, Fraunhofer Institute for Algorithms and Scientific Computing (SCAI), Schloss Birlinghoven, 53757 Sankt Augustin, Germany; Bonn-Aachen International Center for Information Technology (B-IT), University of Bonn, Friedrich-Hirzebruch-Allee 6, Bonn 53113, Germany; Department of Bioinformatics, Fraunhofer Institute for Algorithms and Scientific Computing (SCAI), Schloss Birlinghoven, 53757 Sankt Augustin, Germany; Bonn-Aachen International Center for Information Technology (B-IT), University of Bonn, Friedrich-Hirzebruch-Allee 6, Bonn 53113, Germany; Knowledge Management, German National Library of Medicine (ZB MED)—Information Centre for Life Sciences, Friedrich-Hirzebruch-Allee 4, Bonn 53115, Germany; Graduate School DILS, Bielefeld Institute for Bioinformatics Infrastructure (BIBI), Faculty of Technology, Bielefeld University, Postfach 10 01 31, Bielefeld, Nordrhein-Westfalen 33501, Germany; Information management, Institute of Geodesy and Geoinformation, University of Bonn, Katzenburgweg 1a, Bonn 53115, Germany

## Abstract

MicroRNAs (miRNAs) play important roles in post-transcriptional processes and regulate major cellular functions. The abnormal regulation of expression of miRNAs has been linked to numerous human diseases such as respiratory diseases, cancer, and neurodegenerative diseases. Latest miRNA–disease associations are predominantly found in unstructured biomedical literature. Retrieving these associations manually can be cumbersome and time-consuming due to the continuously expanding number of publications. We propose a deep learning-based text mining approach that extracts normalized miRNA–disease associations from biomedical literature. To train the deep learning models, we build a new training corpus that is extended by distant supervision utilizing multiple external databases. A quantitative evaluation shows that the workflow achieves an area under receiver operator characteristic curve of 98% on a holdout test set for the detection of miRNA–disease associations. We demonstrate the applicability of the approach by extracting new miRNA–disease associations from biomedical literature (PubMed and PubMed Central). We have shown through quantitative analysis and evaluation on three different neurodegenerative diseases that our approach can effectively extract miRNA–disease associations not yet available in public databases.

**Database URL**: https://zenodo.org/records/10523046

## Introduction

Short RNA molecules such as microRNAs (miRNAs) that bind to target messengerRNAs (mRNAs) play important roles in post-transcriptional processes and regulate major cellular functions [1]. Deregulation of expression of miRNAs, which impacts the gene expression patterns and disrupts cellular processes, has been associated with several human diseases such as respiratory diseases [2–4], cancer [1], and Alzheimer’s disease (AD) [5–7]. Targeting disease-associated mRNAs through selected miRNAs makes these molecules interesting candidates for therapy, which is even more of significance with further clinical advancements in miRNA delivering technologies [1]. However, this requires a thorough knowledge of the involvement of specific miRNAs in normal biological processes and in diseases, which is obtained through *in vivo* and *in vitro* experiments and published in research literature.

Extraction of such miRNA–disease associations from the literature can be performed through text mining techniques. In the past, Bagewadi *et al*. [8] proposed the extraction of miRNA, species, genes/proteins, and disease annotations and their relations by creating new corpora and utilizing rule-based methods (such as regular expressions) and machine learning methods (such as support vector machines). They reached an F1-score of up to 76% for miRNA relations. In addition, Li *et al*. [9] created a rule-based text mining system called miRTex that focused on extracting just miRNA–gene and gene–miRNA regulation relations from scientific literature. Their final system achieved an F1-score of 88% on a test set of 150 PubMed abstracts; however, the recall (81%) was significantly lower than precision (96%), which is a common characteristic of a rule-based system. Gupta *et al*. [10] proposed the miRiaD text mining tool, which reached an F1-score of 89.4% on a set of 200 sentences, to extract miRNA–disease relations from the entire Medline identifying 8301 abstracts containing such relations. The tool BeFree, proposed by Bravo *et al*. [11], that exploits morphosyntactic information of the text reached an F1-score of 85% for the extraction of gene–disease associations that also includes miRNAs. The results of BeFree are integrated in the DisGeNET database, a platform for disease genomics [12].

In the meantime, transformer-based general language models such as Bidirectional Encoder Representations from Transformes (BERT) [13] or Generative Pre-trained Transformer (GPT) [14] have revolutionized the field of natural language processing (NLP), as they can effectively represent long-term interactions in text using the built-in attention mechanism [15]. These models are pretrained on large text corpora to model the English language. Furthermore, various biomedical domain-specific models such as BioBERT [16], BioMegatron [17], and ClinicalBERT [18] have been created by additional pretraining on PubMed abstracts, PMC full-text documents, and clinical notes. These biological language models have been proposed for various biomedical NLP (bioNLP) tasks, such as named entity recognition (NER), relation extraction (RE), and document classification. In the past, the bioNLP research has mostly focused on extracting protein–protein interactions [19], drug–drug interactions [20], adverse effects detection [21], clinical entity extraction [22, 23], molecular event extraction [24], and more [25, 26].

In this paper, we introduce a deep learning-based text mining workflow that extracts miRNA–disease associations from the literature. The text mining workflow defines three different tasks: (I) detection of miRNA and disease entities (NER), (II) linking of miRNA and disease entities to specific database identifiers [entity linking (EL)], and (III) detection of their associations (RE). We also create a new training dataset containing miRNA–disease associations using distant learning from multiple databases, which is used to train the relation extraction model. After evaluating the promising prediction performance of our workflow, we use it to extract miRNA–disease associations from PubMed between 2020 and 2023. We further discuss the predicted associations in the context of three diseases of interest. For re-usage, we publish the new corpus, the predicted associations, and the source code of our workflow.

## Materials and methods

First, we describe all datasets that are required for the three tasks NER, EL, and RE. Next, the training, evaluation, and application of machine learning modeling approach are described in detail

### Datasets

#### Collection of miRNA and disease entity recognition datasets

We used the openly available National Center for Biotechnology Information (NCBI) Disease published by NCBI [27] and BioCreative V Chemical Disease Relation (BC5CDR Disease) [28] corpora that both contain disease mention annotations. These annotations also include entity links to concept identifiers from the Medical Subject Headings (MeSH) database, whereas miRNA mentions are included in miRNA [8] and miRTex [9] corpora. For all datasets, we used the so-called Beginning-Inside-Outside-standoff format [29] for labeling the datasets, where ‘O’ is assigned to every token that does not represent an entity, ‘B’ corresponds to the first token of an entity, and ‘I’ is assigned to following tokens of an entity.

#### Building a corpus of miRNA–disease relations using distant supervision

Distant or weak supervision aims to create a training dataset (or corpus) by extracting instances from a single or multiple existing knowledge bases, in order to reduce the amount of manual curation effort [30]. To create a suitable training corpus containing miRNA–disease relations, we used two different databases, namely Human microRNA Disease Database 3 [31, 32] and miR2Disease [33]. We first applied rule-based approaches to extract miRNA and disease entities from PubMed abstracts using MiRNADetector [8] and JProMiner, a re-engineered NER algorithm based on the ProMiner software developed by Hanisch *et al*. [34]. In a postprocessing step, we filtered out sentences with no miRNA or disease annotations. Furthermore, sentences containing multiple miRNA or disease annotations were manually curated. We further extended our corpus with miRNA–disease relations published by [8]. An overview of all datasets used for training can be seen in Table 1, including some descriptive statistics on the number of mentions and relations for each individual dataset.

**Table 1.** Overview of training and test dataset including number of sentences, mentions, and relations in each dataset

		Training		Test	
NER class	Dataset name	Sentences (%)	Mentions (%)	Sentences (%)	Mentions (%)
Disease	NCBI Disease [27]	6224 (87)	5920 (86)	907 (13)	960 (14)
Disease	BC5CDR Disease [28]	9278 (65)	8427 (66)	4950 (35)	4424 (34)
miRNA	miRNA corpus [8]	1864 (70)	528 (58)	780 (30)	375 (42)
miRNA	miRTex corpus [9]	2063 (57)	1540 (56)	1556 (43)	1217 (44)
		**Training relations**		**Test relations**	
**RE class**	**Dataset name**	**Positive (%)**	**Negative (%)**	**Positive (%)**	**Negative (%)**
gene–disease	GAD corpus [11]	2520 (90)	2276 (90)	281 (10)	253 (10)
gene–disease	EU-ADR [53]	235 (90)	83 (90)	27 (10)	10 (10)
miRNA–disease	SCAI-MDC (ours)	1468 (76)	1032 (78)	460 (24)	290 (22)

The numbers in brackets represent the proportions in the training and test sets. The proportions of all external datasets are kept as defined in the original studies. In the case of relation datasets, the number of sentences is identical to the number of relations.

### Training and application of the miRNA–disease detection pipeline

#### General workflow

**Figure 1.**
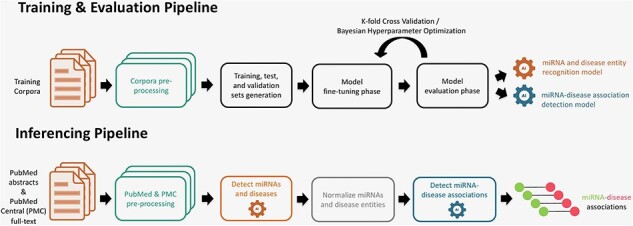
Training, evaluation, and inferencing pipelines for extraction of miRNA and disease entities (NER) and their associations (RE).

The miRNA–disease association detection workflow consists of two pipelines, which are illustrated in Fig. 1. The training and evaluation pipeline is used to train models that are able to detect miRNA and disease entities and their underlying associations between them. The inferencing pipeline is used to apply the trained models to detect miRNA–disease associations from huge text collections.

In the training and evaluation pipeline (Fig. 1), the first step consists of reading and preprocessing the NER and RE corpora. In the next step, we split the whole corpus in various training, validation, and test sets. For NER, we performed tokenization of sentences and prepared the entities and resulting tokens for IOB-tagging. For RE, we also tokenized the sentences and masked the miRNA and disease entities with predefined tokens for further processing. As each model has its own specific wordpiece tokenization scheme, we utilized the model-specific tokenizer that converts the instances into fixed-sized vectors. In the next stage, these instances are used to fine-tune and optimize the pretrained models for both NER and RE tasks. A model evaluation and selection reveals the best models that can be used for prediction. The inferencing pipeline (Fig. 1) is designed to predict associations from text. First, documents from databases [PubMed and PubMed Central (PMC)] are prepared for inferencing. Subsequently, the models for NER and RE are applied to detect miRNA entities, disease entities, and their associations. In a normalization step, the miRNA and disease entities are normalized to the specific database concepts, namely to Mirbase and MeSH identifiers.

#### Fine-tuning of BERT-based models

We used the BioBERT [16] and BioMegatron [17] models for our experiments. Both are based on the BERT model published by Google [13], which is trained in a self-supervised manner on huge amounts of text that were obtained from OpenBooks, Wikipedia, etc. BioBERT and BioMegatron used the pretrained BERT model and its wordpiece tokenizer. Both were trained further using both PubMed and PMC articles to obtain a domain-specific model for biomedicine. BERT, BioBERT, and BioMegatron are so-called general purpose language models that can be used for various text mining tasks such as NER, RE, document classification, or question answering. To use them for these tasks, they need to be further fine-tuned in a supervised manner on datasets that are specific to the underlying tasks.

**Figure 2.**
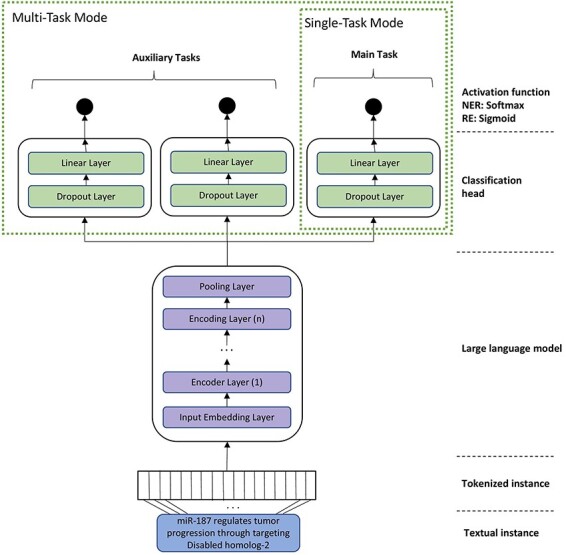
A general architecture of the model for task-specific fine-tuning of domain-specific language models (such as BioBERT and BioMegatron). The STM contains just one head. MTM contains additional heads for each auxiliary task or corpus.

For RE, we experimented with two different training modes, namely single-task mode (STM) and multi-task mode (MTM). In the STM, the models were fine-tuned on a single dataset, whereas in MTM, related datasets were used for fine-tuning the various classification heads of the BioBERT model. In MTM, we apply the paradigm of multi-task learning, where a single model is trained to accomplish multiple closely-related tasks simultaneously by using a shared representation [35]. Previous studies have shown that multi-task learning can be beneficial as it improves the generalization by focusing on the commonalities of the tasks and learning relevant features contained in training data of different tasks [35]. The architecture of the final model that is used for fine-tuning BioBERT and BioMegatron is depicted in Fig. 2. We also experimented with different variants for the classification head (such as multiple linear layers, bottleneck architecture). However, the experiments revealed that a simple linear layer works best in all cases. Therefore, our final model contains a single linear layer on top of the pre-trained BioBERT and BioMegatron models.

#### Linking of miRNA and disease entities

We implemented a rule-based system to link miRNA entities to miRBase identifiers. miRBase [36] is a database that includes published miRNA sequences and annotations, and furthermore, it provides a registry with unique names for miRNAs. To link the recognized disease entities to MeSH identifiers, we used the software NormCo [37].

#### Evaluation of NER and RE models

For NER, we used precision, recall, and F_1_-measure to determine the performance of the models. For RE, which is defined as a binary classification, we used the area under receiver operator characteristic curve (AUROC) and precision–recall curve (AUPR) to evaluate performance. We also provide a confusion matrix report for tasks where it is appropriate, which includes true positive, false positive, false negative, and true negative cases.

In an initial stage, we prepared training and test splits for each dataset. It is important to note that the proportions of the splits are kept as defined in their original studies. Furthermore, we applied five-fold cross-validation to choose the best models. For each iteration, we created a stratified split of the training dataset into training (*n* − 1 folds) and validation (1 fold) datasets. We then trained on the training dataset and evaluated (and optimized the hyperparameters) on the validation dataset for *n* iterations. The results of *n* evaluations are aggregated and the standard deviation is reported. The final evaluation of the best models was performed on the withheld independent test set.

#### Hyperparameter optimization

We performed a Bayesian hyperparameter optimization [38] using the Optuna [39] framework for all our models with the appropriate training data. We assessed the intermediate and final performances of each experimental trial using the F_1_-measure (NER) and AUROC (RE). The results were captured in an SQL database for later analyses, such as identifying the best experimental trials. The captured trial data were also used by the Optuna pruner to identify and halt unpromising trials already at an early stage.

### Comparison of predicted associations with DisGeNET

In a consecutive analysis, we compare our predicted associations with data from DisGeNET, where we focus on three different diseases, namely epilepsy, AD, and Parkinson’s disease (PD). To compare the associations, it was necessary to retrieve MeSH and UMLS concept identifiers for the disease terms as our workflow normalizes to MeSH and DisGeNet include UMLS identifiers. To retrieve the MeSH and UMLS classes for these diseases, we first gathered all subclasses of the disease from the MONDO ontology [40] and then retrieved their MeSH and UMLS associated identifiers. Both tasks were performed using the OLS4 API (https://www.ebi.ac.uk/ols4). After gathering the associations, we filtered them using the disease identifiers.

## Results

### Detection of miRNA and disease entities

To detect miRNA and disease mentions, we used the pretrained BioBERT and BioMegatron models and fine-tuned them on various datasets. To identify the best possible model variant based on the training data, we employed a 5-fold cross-validation during training. Based on the performance assessed through cross-validation, we used the optimized hyperparameters to train the final model on the whole training dataset. The generalization performance of the final models was assessed on the held-out test set. Table 2 presents the classification scores for each dataset in the specific test set.

**Table 2.** Evaluation results of NER task models trained and tested on various datasets.

		BioBERT			BioMegatron		
Entity class	Dataset	Prec.	Recall	F_1_	Prec.	Recall	F_1_
Disease	NCBI Disease	84.62	90.09	87.27	**88.22**	**91.25**	**89.71**
	BC5CDR	82.07	85.39	83.70	**85.49**	**87.75**	**86.60**
	NCBI Disease + BC5CDR	–	–	–	**86.26**	**87.83**	**87.04**
miRNA	miRNA	91.32	**98.13**	94.60	**91.75**	9787	**94.71**
	miRTex	93.93	95.79	94.85	**96.59**	**97.62**	**97.10**
	miRNA + miRTex	–	–	–	**94.51**	**96.23**	**95.36**

The confusion matrix of the BioMegatron model is included in Supplementary Table S7. – indicates data are not available. Bold entries represent the best results.

For the NCBI dataset, we achieved the highest performance with an F1-score of 89.71%, precision of 88.22%, and recall of 91.25%. For the BC5CDR dataset, the best F1-score was 86.60% with precision of 85.49% and recall of 87.75%. We also trained a model with the combination of both datasets, where a micro F1-score of 87.04% was reached on the combined test set. Overall, BioMegatron performed better than BioBERT, which is probably due to the large parameter size of the BioMegatron model.

In the case of miRNA entity detection, the best F1-measure for the miRNA dataset was 94.70%, precision was 91.75%, and recall was 97.86%, and the best performance for the miRTex dataset was achieved with an F1-score of 97.10% and a precision of 96.59%. The training on the combined dataset reached a micro F1-score of 95.36%. Similar to the disease category, the BioMegtron model performed significantly better than BioBERT, while the BioBERT model delivered the best recall of 98.13% on the miRNA dataset. The confusion matrices of the best NER models are provided in Supplementary Table S7. The optimized hyperparameters of the best NER models are included in Supplementary Table S1–S4 and S6.

We also experimented with MTM; however, the results were not significantly better in comparison to the STM. Although the NER datasets and tasks share with each other certain similarities, the significant differences in the annotation guidelines and their varying levels of complexity of the mentions likely reduced the effectiveness of the multi-task approach. The shared representations in the model might have led to negative transfer, showing a drop in the model performance. Similar observations have also been made by Crichton *et al*. [41]. Hence, for further analysis we focused only on STM.

It is important to note that the BC5CDR corpus is a sub-corpus of the CTD-Pfizer corpus [42]. The creators of the corpus aimed to investigate the potential involvement of pharmaceutical drugs in cardiovascular, neurological, renal and hepatic toxicity. Therefore, the BC5CDR corpus is focused on drugs and their role in toxicity [42]. In contrast, the NCBI disease corpus is intended to represent the entire PubMed. In an analysis of both corpora performed by Kühnel and Fluck [43], they revealed that the BC5CDR corpus contains more complex contexts, including abbreviations from diseases but also mentions several gene names, such as *BRCA1*, resembling the structure of an abbreviation. This could explain why the model performances for the NCBI Disease dataset are slightly better.

### Detection of miRNA–disease associations

We trained an association detection model using the BioMegatron model on our own training dataset (80% of the whole dataset). As BioMegatron, in comparison to BioBERT, delivered the best results in almost all cases, we only focused on experimenting with the BioMegatron model further. The model selection was based on five-fold cross-validation. After choosing the best hyperparameters, we evaluated the final model performance using measures, such as AUROC and AUPR on an independent test set (20% of the whole dataset). Table 3 illustrates the evaluation performances. We reached a high rate of 9758% AUROC and 9755% AUPR with the STM. Even higher scores are reached with the MTMs, amounting to 98.02% and 98.66% for AUROC and AUPR, respectively. The receiver operator characteristic and precision–recall curves of the best model are depicted in Supplementary Figure S1. The optimized hyperparameters of the best RE model are included in Supplementary Tables S5 and S6.

**Table 3.** Evaluation results of RE task on test dataset for STM and MTM training modes based on BioMegatron model.

Datasets	Mode	AUROC (in %)	AUPR (in %)
miRNA–disease	STM	97.58	97.55
	MTM	**98.02**	**98.66**

Bold entries represent the best results.

### Prediction of miRNA–disease associations from PubMed

**Figure 3.**
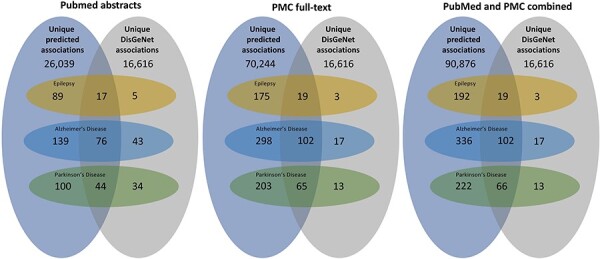
An overview of the total predicted unique associations between miRNA and diseases in comparison to the DisGeNET database. The three subfigures represent the results extracted between 2020 and 2023 from PubMed abstracts, PMC full-text documents, and both combined. Furthermore, it provides an overview over the miRNA–disease associations of three diseases (epilepsy, AD, and PD).

We applied our miRNA–disease association extraction workflow on around 6.1 million PubMed abstracts and 1.98 million PMC full-text documents published between 2020 and 2023. Overall, the workflow predicted 727 009 (unique: 75 887) normalized positive associations found in 69 816 PMC and PubMed documents. These associations include 2730 disease and 2427 miRNA concepts. Overall, 374029 positive associations (unique: 52624; found in 59187 PMC documents) of them have a high confidence score of 90% (retrieved through a sigmoid function). Figure 3 provides an overview of the total predicted miRNA–disease associations for PubMed abstracts, PMC full-text documents, and both corpora combined.

In a subsequent analysis, we filtered for associations of three different diseases, namely epilepsy, AD, and PD (see Fig. 3). For epilepsy, AD, and PD, the workflow detected 2226 (unique: 211), 6306 (unique: 438), and 3159 (unique: 287) miRNA–disease associations, respectively. In a first step, we compared the extracted miRNA associations with those in the existing database DisGeNET, which contains curated miRNA–disease associations extracted from different resources before 2020. In all cases, we could significantly increase the number of miRNA–disease associations and found a high number of new relations not contained in DisGeNET (see Fig. 3). Since we focus on new findings, only a small number of the relations overlap with the relations contained in DisGeNet and others are only available in DisGeNet.

We also performed an analysis of the missed DisGeNet associations for the year 2020 of the three diseases. In total, DisGeNet contains four unique miRNA–disease associations for epilepsy, eight for AD, and eight for PD from publications published in the year 2020. Only two associations (one for AD and one for PD) were missed by our workflow. In these cases, the workflow predicted wrong association labels (no association). All the other missed associations were from publications published before 2020, which we have not included in our workflow. To expand this analysis, we randomly analyzed additional unique associations that were missed by our pipeline. In some cases, the association was detected, however, with a lower score (<0.9). In other cases, the disease and miRNA normalizer were not able to properly normalize the mentions. We provide some examples of these cases in the Supplementary Section ‘Examples of Workflow Issues’.

#### Evaluation of newly detected miRNA–disease associations

For all three diseases, AD, PD, and epilepsy, we randomly choose associations from the predicted results of the PubMed corpora that had a high score (>0.9). Examples of extracted associations with their corresponding sentences are shown in Table 4. For AD, our workflow detected three miRNA–disease associations from a study by Kumar *et al*. [5]. In this study, by analyzing postmortem brains of AD and control samples using a miRNAs microarray platform, the authors have addressed the question of whether synaptosomal miRNAs affect AD synapse activity. They found that three specific miRNAs are potentially associated with AD Braak stages. In the case of PD, our workflow detected two miRNA–disease associations from the study published by Chen *et al*. [44]. The authors investigated blood circulating miRNAs that are proposed to be promising biomarkers for neurodegenerative diseases such as PD. They analyzed the plasma of PD patients, multiple system atrophy patients, and healthy controls. Our workflow detected two associations from the study [45], where the authors studied the role of let-7b miRNAs in temporal lobe epilepsy (TLE). They found a novel noncoding RNA-mediated mechanism involving the miRNA let-7b and H19 [a long noncoding RNA (lncRNA)] in seizure-induced glial cell activation.

**Table 4.** Examples of predicted miRNA–disease associations for AD, PD, and epilepsy with their corresponding sentences.

Disease	miRNA	Sentence	PMID
AD	hsa-miR-501-3p (MIRBASE:MIMAT0004774)	The **miR-501-3p**, miR-502-3p, and miR-877-5p were identified as potential synaptosomal miRNAs upregulated with disease progression based on **AD** Braak stages.	36454178
AD	hsa-miR-502-3p (MIRBASE:MIMAT0004775)	The miR-501-3p, **miR-502-3p**, and miR-877-5p were identified as potential synaptosomal miRNAs upregulated with disease progression based on **AD** Braak stages.	36454178
AD	hsa-miR-877-3p (MIRBASE:MIMAT0004949)	The miR-501-3p, miR-502-3p, and **miR-877-5p** were identified as potential synaptosomal miRNAs upregulated with disease progression based on **AD** Braak stages.	36454178
PD	hsa-miR-133b (MIRBASE:MIMAT0000770)	Elevated **miR-133b** and miR-221-3p distinguished **PD** from controls with 84.8% sensitivity and 88.9% specificity.	34315950
PD	hsa-miR-221-3p (MIRBASE:MIMAT0000278)	Elevated miR-133b and **miR-221-3p** distinguished **PD** from controls with 84.8% sensitivity and 88.9% specificity.	34315950
Epilepsy	hsa-let-7b-5p (MIRBASE:MIMAT0000063)	Overexpression of **let-7b** inhibited hippocampal glial cell activation, inflammatory response and **epileptic seizures** by targeting Stat3.	32648622
Epilepsy	hsa-let-7b-5p (MIRBASE:MIMAT0000063)	LncRNA H19 could competitively bind to **let-7b** to promote hippocampal glial cell activation and epileptic seizures by targeting Stat3 in a rat model of **TLE**.	32648622

The normalized miRNA names mentioned in the second column corresponds to the bold miRNA names in the Sentence column.

For a systematic analysis of the newly found associations, we analyzed the precision and recall for the PD–miRNA associations. To check the overall precision of the newly predicted associations, 30 sentences were examined. This analysis showed that only two extractions were incorrect. In the sentence ‘PD was associated with postoperative expression of GFAP; ePOCD was associated with postoperative expression of microRNA-21-5p and GFAP as well as intraoperative expression of NSP’ (PMID:34 300 256) [46], the abbreviation ‘PD’ means postoperative delirium and hence the association with the disease PD is incorrect. The second error occurred in an extraction from the text fragment ‘[…] Mitochondrial complex I deficiency and functional abnormalities are implicated in the development of PD. MicroRNA-29a […]’ (PMID: 36 174 668) [47] that consists of more than one sentence and could not be verified as the correct source for the extracted association, although the relation was mentioned later in the abstract. In summary, this analysis shows a precision of over 93%.

In order to analyze the recall, we utilized a systematic review of PD–miRNA associations published by [48]. All referenced associations from publications in 2020 to 2023 were compared with our automatically extracted set. Out of 23 associations, a total of 15 associations were extracted from the same abstract also referenced by the review, but 8 could not be found in the abstracts. These eight associations were reviewed further. Two associations (from Wu 2020 [49]) could not appear in our result set as the corresponding publication journal ‘Acta Medica Mediterranea’ is not part of the Medline. Furthermore, in the publication by Ravanidis et al. [50], the two associations were not mentioned in the abstract, but only in the full-text. Finally, in the publication by Cressati et al. [51] , miR-153 and miR-223 were mentioned in the abstract and these associations were correctly recognized, but in the review, they are listed as miR-153-3p and miR-223-5p. Only two associations were not found by the automated extraction system although they were mentioned in the abstract. These were missed because the corresponding miRNAs were not recognized. In summary, this analysis shows that 19 associations were described in the Medline articles, of which our system recognized 17 associations. This corresponds to a recall of 89%.

This evaluation shows that even after the sequential execution of automated NER, entity linkage, and association recognition, which have their own error rate that adds up in the overall result, the performance of the automated extraction system is remarkable and therefore very well suited to support systematic reviews such as that published for PD by Guévremont *et al*. [48].

## Discussion

In this work, we presented a workflow for automatically extracting miRNA–disease associations from vast unstructured literature. The workflow is based on a large language model fine-tuned on a new corpus generated using a distant supervision technique. Due to the pretraining of large language model (for e.g. BioMegatron) on a huge corpora and the integrated self-attention mechanism, the model can exploit semantic and syntactic aspects of sentences and incorporates local contextual features of the included entities to extract relations with high accuracy. We used the workflow to extract miRNA–disease associations from Medline abstracts and analyzed the extracted set for AD, PD, and epilepsy. Compared to the existing curated database DisGeNet, where miRNA–disease associations were provided until 2020, we extracted a high number of new associations from Medline abstracts for the years 2020–23. An independent evaluation of the newly extracted PD–miRNA associations showed that we achieved high precision and high recall with this extraction workflow.

A current limitation of the corpus, and thus of the extraction workflow, is that the associations are encoded and recognized at sentence level. As authors may describe miRNA–disease relations beyond sentences in their publications, the workflow may miss these relations. Nevertheless, the evaluation showed that at least for PD, the PD–miRNA relations are usually expressed in the same sentence in abstracts. We missed associations due to false negative disease and miRNA recognition or because the relations were only expressed in the full-text tables. Strategies such as active learning might help to significantly reduce the curation effort to extend the corpus required for training a model that can perform extraction from tables included in full-text documents.

Although the large language models that are specifically designed for the biomedical domain produced great results in our work, incorporating the extensive prior knowledge on miRNAs and diseases directly in large language models might help to improve the results even further. Studies have shown that the process of knowledge fusion is able to overcome the limitations of individual sources by focusing on diverse knowledge. Information such miRNA sequences, disease embeddings obtained from ontologies (such as Disease ontology, MONDO) can be merged with the embeddings from large language models. Also, embeddings obtained through training of graph neural networks on sources such as DisGeNet can be further employed to improve the models. In addition, it might be interesting to combine the literature-based models with new prediction models learning feature embeddings for miRNAs and diseases through graph machine learning [52].

In summary, by automatically generating a training corpus using distance learning methods and training a model based on a state-of-the-art large language model, we have demonstrated the promising performance of our trained workflow. Our evaluation results based on PD-miRNA associations strongly suggest that our workflow can provide useful support for extracting miRNA–disease relations.

## Conclusion

In this work, we proposed a well-performing large language model approach for the identification of miRNA–disease relations from biomedical literature. The approach consists of modules that can perform the detection of miRNA and disease mentions, as well as the identification of their relationship. In order to extend the miRNA–disease training corpora, we applied the distant supervision technique using multiple publicly available databases. In our experiments with multiple state-of-the-art large language models, BioMegatron performed the best for the extraction of miRNA–disease associations. A high number of new associations could be identified with a high level of precision of recall and precision, when applying the whole machinery to infer associations from biomedical literature between 2020 and 2023.

The creation and use of dedicated databases that can contain many types of relations is considered best practice in biomedical research and up-to-date information is in high demand. However, to keep these databases up to date with the current scientific advancements is a major challenge. The solution is often to establish collaborations with researchers and institutions to provide regular updates. However, this requires a huge amount of human effort. This creates a demand for automated data mining techniques that should always be employed to extract relevant information from scientific literature and update the databases accordingly. With the three different case studies on neurodegenerative diseases such as AD, for which we identified and discussed novel relations that are yet missing in databases such as DisGeNet, we demonstrated the applicability and feasibility of our workflow for retrieving novel, hidden relations from literature.

Automated techniques for information extraction need to be regularly revised to keep up with the pace of development in NLP. Recent large language models such as ChatGPT, BARD, some of which are unfortunately not yet available for scientific experimentation, open up new avenues for solving challenges. Future studies are required to find out exactly how these models can be utilized to not only extract a single type of relation but also to solve many complex bioNLP challenges at once.

## Supplementary Material

baae066_Supp

## Data Availability

We provide our code at https://github.com/SCAI-BIO/mirna-disease-association-detection. Our database is located at https://zenodo.org/records/10523046
