# Supplementary material for: Dataset of miRNA–disease relations extracted from textual data using transformer-based neural networks
Source: Database (Oxford). 2024 Aug 5;2024:baae066. doi: 10.1093/database/baae066 (PMC11300841; doi:10.1093/database/baae066)
Supplement: baae066_Supp [file baae066_supp.zip › suppl_data/Supplementary - Dataset of miRNA-Disease Relations Extracted from Textual Data using Transformer-based Neural Networks.docx]

Supplementary for Dataset of miRNA-Disease Relations Extracted from Textual Data using Transformer-based Neural Networks

**Sumit Madan^1,*^, Lisa Kühnel^2,3^, Holger Fröhlich^1,4^, Martin Hofmann-Apitius^1,4^, and Juliane Fluck^2,3,5^**

^1^ Department of Bioinformatics, Fraunhofer Institute for Algorithms and Scientific Computing (SCAI), Schloss Birlinghoven, 53757 Sankt Augustin, Germany

^2^ German National Library of Medicine (ZB MED) - Information Centre for Life Sciences, 53115 Bonn, Germany

^3^ Graduate School DILS, Bielefeld Institute for Bioinformatics Infrastructure (BIBI), Faculty of Technology, Bielefeld University, 33615 Bielefeld, Germany

^4^ Bonn-Aachen International Center for Information Technology (B-IT), University of Bonn, 53113 Bonn, Germany

^5^ Institute of Geodesy and Geoinformation, University of Bonn, 53115 Bonn, Germany

^*^ Corresponding authors: [sumit.madan@scai.fraunhofer.de](mailto:sumit.madan@scai.fraunhofer.de)

# Section S1. Hyperparameter Optimization Results

| **Hyperparameter** | **Final model values** |
| --- | --- |
| Adam epsilon | 1.079057e-09 |
| Classification head dropout probability | 0.5 |
| Weight decay | 8.4972893e-08 |
| Gradient accumulation steps | 1 |
| Learning rate | 1.8375e-05 |

Table S1. Optimized hyperparameters of the BioMegatron NER model trained on NCBI Disease dataset

| **Hyperparameter** | **Final model values** |
| --- | --- |
| Adam epsilon | 1.609e-07 |
| Classification head dropout probability | 0.6 |
| Weight decay | 0.0002077496 |
| Gradient accumulation steps | 2 |
| Learning rate | 3.67977e-05 |

Table S2. Optimized hyperparameters of the BioMegatron NER model trained on BC5CDR dataset

| **Hyperparameter** | **Final model values** |
| --- | --- |
| Adam epsilon | 2.172e-07 |
| Classification head dropout probability | 0.5 |
| Weight decay | 3.0914e-06 |
| Gradient accumulation steps | 1 |
| Learning rate | 4.09604e-05 |

Table S3. Optimized hyperparameters of the BioMegatron NER model trained on miRNA dataset

| **Hyperparameter** | **Final model values** |
| --- | --- |
| Adam epsilon | 8.88186e-10 |
| Classification head dropout probability | 0.5 |
| Weight decay | 4.604589e-09 |
| Gradient accumulation steps | 2 |
| Learning rate | 3.7362e-05 |

Table S4. Optimized hyperparameters of the BioMegatron NER model trained on miRTex dataset

| **Hyperparameter** | **Final model values** |
| --- | --- |
| Adam epsilon | 1.0457e-10 |
| Classification head dropout probability | 0.5 |
| Weight decay | 0.0022353644 |
| Gradient accumulation steps | 4 |
| Learning rate | 3.37329e-05 |

Table S5. Optimized hyperparameters of the RE multi-task model based on BioMegatron

| **Hyperparameter** | **Model values** |
| --- | --- |
| Dimensionality of the encoder layers, pooler layers, and classification head layer | 1024 |
| Dimensionality of the feed forward layer in encoder | 4096 |
| Layer normalization epsilon | 1e-12 |
| Number of attention heads | 16 |
| Number of hidden layers | 24 |
| Vocabulary size | 29056 |
| Dropout probabilities of hidden and attention layers | 0.1 |
| Maximum position embeddings | 512 |
| Optimizer | AdamW |
| Adam beta 1 | 0.9 |
| Adam beta 2 | 0.999 |
| Batch size | 8 |
| Warm up steps | 100 |

Table S6. Further fixed hyperparameters for training of model EMBO/BioMegatron345mCased (<https://huggingface.co/EMBO/BioMegatron345mCased>).

# Section S2. Confusion Matrix of NER Models

| **Entity class** | **Dataset** | **True Positive** | **False Positive** | **False Negative** |
| --- | --- | --- | --- | --- |
| Disease | NCBI Disease | 876 | 117 | 84 |
|  | BC5CDR | 3,882 | 659 | 542 |
|  | NCBI Disease + BC5CDR | 4,729 | 753 | 655 |
| miRNA | miRNA | 367 | 33 | 8 |
|  | miRTex | 1,188 | 42 | 29 |
|  | miRNA + miRTex | 1,532 | 89 | 60 |

Table S7. Confusion matrix of NER models only for BioMegatron model.

# Section S3. Receiver Operator Characteristic (ROC) and Precision Recall Curves of Best miRNA-Disease Association Model


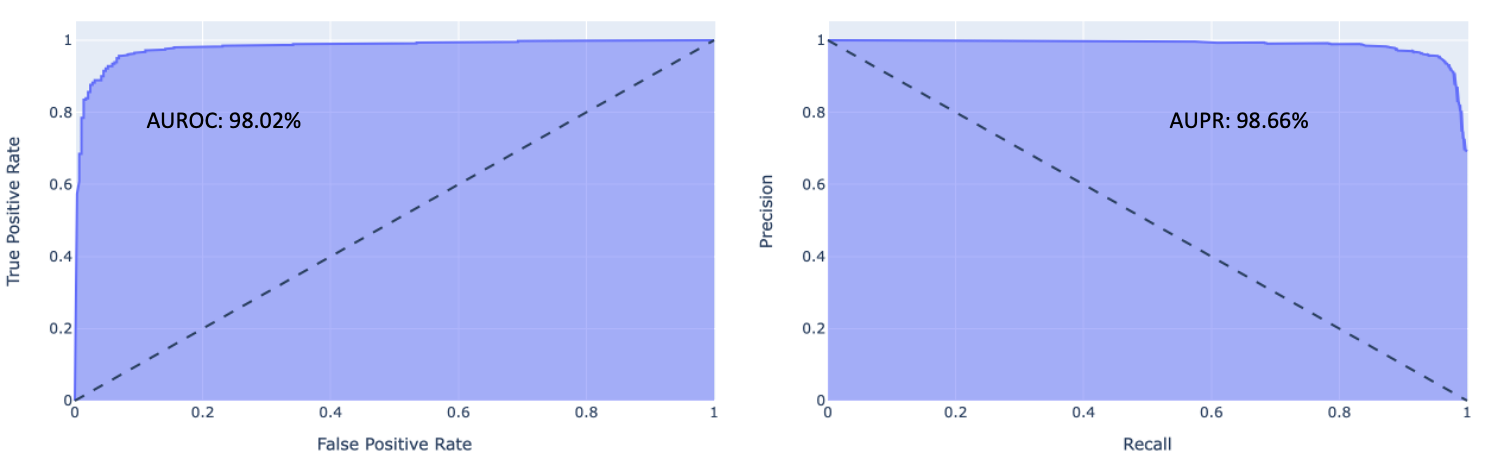


Figure S1. Receiver operator characteristic (ROC) curve (left) and precision-recall curve (right) obtained by applying the best model on unseen test data.

# Section S4. Examples of Workflow Issues

1. Issue: workflow detected real association as “no association” (false negative):
   1. Example:

PMID:31766078,

MiRNA mention: miR-563,

Disease mention: Parkinson’s disease,

Sentence: “Previous investigations have revealed that miR-563 is associated with a number of diseases including the ossification of posterior longitudinal ligament, Parkinson's disease or drug resistance to leukemia.”

- 1. Example

PMID:31685236,

MiRNA mention: miR-6840-5p,

Disease mention: AD (Alzheimer’s disease)

Sentence: “Although, overall, we did not identify a strong effect of AD GWAS variants on miRNA expression in this cell type, we highlight 2 notable outliers, that is, miR-29c-5p and miR-6840-5p.”

1. Issue: workflow detected association with lower score than 0.9:
   1. Example

PMID:31797701

MiRNA mention: miR-3922-5p

Disease mention: Breast cancer

Sentence: 'Conclusions: The novel lncRNA HOXC-AS3 acts as a miR-3922-5p sponge to upregulate PPP1R1A protein expression, and thus results in promoting breast cancer metastasis.',

Score: 0.409

1. Issue: disease normalization couldn’t normalize the term ‘BC’ to breast cancer concept
   1. Example

PMID:31721061

MiRNA mention: miR-302b

Disease mention: BC (breast cancer)

Sentence: ‘The present study suggests that miR-302b functions as a tumor suppressor in BC and inhibits the tumor progression of BC via targeting RUNX2.'

1. Issue: non-identical identifiers produced by our miRNA normalization component. DisGeNet normalized miR-7-5p to NCBI Gene ID:407044 and our miRNA normalization component produced NCBI Gene ID:407045. However, in this case both identifiers are valid.
   1. Example

PMID:31635802

MiRNA mention: miR-7-5p (normalized to MIRBASE:MIMAT0000252 and NCBI Gene ID:407045)

Disease: breast cancer

Sentence: The reduction of miR-7-5p was required in the augmentation of breast cancer development induced by lnc-LUCAT1 over-expression.
